# Three New Genetic Loci (R1210C in *CFH*, Variants in *COL8A1* and *RAD51B*) Are Independently Related to Progression to Advanced Macular Degeneration

**DOI:** 10.1371/journal.pone.0087047

**Published:** 2014-01-31

**Authors:** Johanna M. Seddon, Robyn Reynolds, Yi Yu, Bernard Rosner

**Affiliations:** 1 Ophthalmic Epidemiology and Genetics Service, Tufts University School of Medicine and Tufts Medical Center, New England Eye Center, Boston, Massachusetts, United States of America; 2 Department of Ophthalmology, Tufts University School of Medicine, Boston, Massachusetts, United States of America; 3 Channing Laboratory, Brigham and Women's Hospital and Harvard School of Public Health, Harvard University, Boston, Massachusetts, United States of America; Eye Hospital, Charité, Germany

## Abstract

**Objectives:**

To assess the independent impact of new genetic variants on conversion to advanced stages of AMD, controlling for established risk factors, and to determine the contribution of genes in predictive models.

**Methods:**

In this prospective longitudinal study of 2765 individuals, 777 subjects progressed to neovascular disease (NV) or geographic atrophy (GA) in either eye over 12 years. Recently reported genetic loci were assessed for their independent effects on incident advanced AMD after controlling for 6 established loci in 5 genes, and demographic, behavioral, and macular characteristics. New variants which remained significantly related to progression were then added to a final multivariate model to assess their independent effects. The contribution of genes to risk models was assessed using reclassification tables by determining risk within cross-classified quintiles for alternative models.

**Results:**

Three new genetic variants were significantly related to progression: rare variant R1210C in *CFH* (hazard ratio (HR) 2.5, 95% confidence interval [CI] 1.2–5.3, P = 0.01), and common variants in genes *COL8A1* (HR 2.0, 95% CI 1.1–3.5, P = 0.02) and *RAD51B* (HR 0.8, 95% CI 0.60–0.97, P = 0.03). The area under the curve statistic (AUC) was significantly higher for the 9 gene model (.884) vs the 0 gene model (.873), P = .01. AUC’s for the 9 vs 6 gene models were not significantly different, but reclassification analyses indicated significant added information for more genes, with adjusted odds ratios (OR) for progression within 5 years per one quintile increase in risk score of 2.7, P<0.001 for the 9 vs 6 loci model, and OR 3.5, P<0.001 for the 9 vs. 0 gene model. Similar results were seen for NV and GA.

**Conclusions:**

Rare variant *CFH* R1210C and common variants in *COL8A1* and *RAD51B* plus six genes in previous models contribute additional predictive information for advanced AMD beyond macular and behavioral phenotypes.

## Introduction

There is a strong genetic component and an important environmental influence on the development of age-related macular degeneration (AMD) [1], [2]. Common loci in genes in the complement and lipid pathways have been confirmed in several studies. Since 2011, associations with AMD have been shown for a novel rare variant (R1210C) in *CFH* [*3*] common variants in *COL10A1* [*4]*–*[6]* *, COL8A1* [*4]*–*[6*], *VEGFA* [*5*] *,* and *TNFRSF10A* [*6]*, *[7*] Additional common variants were also identified in our international consortium effort based on meta-analyses of several genome-wide case-control association studies. [6] All of these recently reported genes have been shown to be related to advanced AMD when compared with controls. However, the relative impact of most of these new genes on AMD progression has not been reported, and their independent effects, while controlling for all established genetic and non-genetic risk factors for progression, are unknown.

In this study we, therefore, determined: 1) the independent effect of each new genetic locus on AMD progression after controlling for the known genetic, demographic, behavioral and ocular risk factors related to progression, when each new locus was considered separately, 2) whether the new genetic loci related to AMD progression based on this initial analyses remained significant predictors of progression when combined with variables in our previous risk models, and 3) if genes and number of genes significantly add to risk models for AMD progression.

## Methods

The Age-Related Eye Disease Study included a randomized clinical trial to assess the effect of antioxidant and mineral supplements on risk of AMD and cataract and a longitudinal study of AMD that ended in December 2005 [8] Research adhered to the tenets of the Declaration of Helsinki. The research protocol was approved by institutional review boards and all participants signed consent statements. Follow-up time ranged from 0.5 to 13.0 years (mean 8.8 years). Phenotype data were accessed through the Database of Genotypes and Phenotypes (dbGaP). Participants were classified using the Clinical Age-Related Maculopathy Staging System (CARMS) [9], based on ocular examination and grading of fundus photographs at baseline, into 5 stages: normal or stage 1 in both eyes (essentially free of age-related macular abnormalities or a few small drusen), early AMD or stage 2 in the worse eye (mild changes including multiple small drusen, nonextensive intermediate drusen, and/or pigment abnormalities), intermediate AMD or stage 3 in the worse eye (drusen 125 microns or greater diameter, extensive intermediate drusen), stage 4 in one eye (advanced dry AMD with central or non-central geographic atrophy (GA), and stage 5 with advanced neovascular (NV) AMD in one eye at baseline. Both cohorts were classified using this system. Since category 3 patients in the original AREDS classification included non-central geographic atrophy and category 4 included both advanced forms of AMD as well as visual loss regardless of phenotype [8], we reclassified these groups independent of visual acuity level into CARMS grades 4 (GA) and 5 (NV) as described above.

Maximum drusen size within the grid (a 3000 micron radius centered on the fovea) at baseline was used to assess drusen phenotypes for eyes without advanced AMD. Drusen size was based on standard circles with diameters corresponding to 63 µm, 125 µm, and 250 µm. Drusen size was divided into the following categories: <63 µm, 63–124 µm, 125–249 µm, and ≥250 µm. AMD status and drusen size in eyes without AMD at baseline were evaluated.

Progression was defined as either eye progressing from a stage 1, 2 or 3 to either stage 4 or stage 5 at any follow-up visit to the end of the study within each individual. In a subgroup analysis we classified progressors to each advanced stage of AMD separately as progression to GA and progression to NV. Time to progression was recorded for the first eye to progress if both eyes were at risk, and for the fellow eye if one eye was at risk. Individuals were considered progressors if a) there was no advanced AMD in either eye at baseline and they developed advanced AMD in at least one eye during follow-up, or b) they had advanced AMD in one eye at baseline and progressed to advanced AMD in the fellow eye during follow-up. For subjects in group “a” above, we controlled for baseline grade in each eye and evaluated the time to progression in each eye and used the earlier of the two progression times if both eyes progressed at different times. For subjects in group “b” above, we controlled for AMD category in the affected eye at baseline (i.e., CARMS grades 4 and 5), AMD grade in the non-advanced eye at baseline, and evaluated the time to progress in the fellow eye. Demographic and risk factor data, including education, smoking history, and body mass index (BMI), were obtained at the baseline visit from questionnaires and height and weight measurements.

### Genotype Data

Common single nucleotide polymorphisms (SNPs) associated with AMD were evaluated 1) Complement Factor H (*CFH*) Y402H (rs1061170) [10], 2) *CFH* rs1410996, an independently associated single nucleotide polymorphism (SNP) variant within intron 14 of *CFH* [*11*], 3) *ARMS2/HTRA1* (rs10490924) [12]–[14], 4) Complement component 2 or *C2* E318D (rs9332739) [11], [15], 5) Complement Factor B or *CFB* R32Q (rs641153) [11], [15], 6) Complement component 3 or *C3* R102G (rs2230199) [16], [17], 7) complement factor I, or *CFI* (rs10033900), an independently associated SNP located in the linkage peak region of chromosome 4, 2781 base pairs upstream of the 3′ untranslated region of *CFI* [*18*] *,* and 8) hepatic lipase C, or *LIPC* (rs10468017), a promoter variant on chromosome 15q22 [4], 9) Cholesteryl ester transfer protein, or *CETP* (rs3764261) [4], [19], 10) ATP-binding cassette subfamily A member 1, or ABCA1 (rs1883025) [4], [19] 11) TIMP metalloproteinase inhibitor 3, or *TIMP3* (rs9621532) [4], [19], 12) Collagen type VIII, alpha1 or *COL8A1*(rs13095226) [4], [5], 13) *FRK/COL10A1* rs199930 [4], [5], 14) Collagen, type X, alpha 1 or *COL10A1* (rs1064583) [5], [6], 15) Vascular endothelial growth factor A, or *VEGFA* (rs943080) [5], 16) Tumor necrosis factor receptor superfamily, member 10a or *TNFRSF10A* (rs13278062 [6], [7], 17) Apolipoprotein E and Apolipoprotein C- I or *APOE/APOC1* rs4420638 [6], [20], [21], 18) Discodin domain receptor tyrosine kinase 1, or *DDR1* (rs3094111) [6], 19) Solute carrier family 16, member 8 (monocarboxylic acid transporter 3or *SLC16A8* (rs8135665) [6], 20) Transforming growth factor, beta receptor 1 or *TGFBR1* (rs334353) [6], 21) RAD51 homolog B (S. cerevisiae) or *RAD51B* (rs8017304) [6], 22) ADAM metallopeptidase with thrombospondin type 1 motif [6]; ADAMTS9 antisense RNA 2 (non-protein coding) or *ADAMTS9/ADAMTS9-AS2* (rs6795735) [6] 23) Beta 1,3- galactosyltransferase -like or B3GALTL (rs9542236) [6]. The rare variant in *CFH*, R1210C rs121913059 was also evaluated [3].

### Statistical Analyses

Analyses were performed using the Cox proportional hazards model to evaluate relationships between progression of AMD and the following variables: genotypes, age (<65, 65–74, 75+), gender, education (high school or less, more than high school), cigarette smoking (never, past, current), and BMI, which was calculated as the weight in kilograms divided by the square of the height in meters (<25, 25–29.9, and 30+), baseline stage of AMD and drusen characteristics in both eyes. Hazard ratios (HRs) and 95% confidence intervals (CI) were calculated for demographic, behavioral, ocular and genetic factors. The method for calculating the AMD progression risk score and gene risk score based on regression coefficients of all demographic, environmental, genetic and ocular factors, has been reported previously [22]–[24].

Survival analysis was used to determine 5-year and 10-year cumulative incidence rates of AMD and the advanced AMD subtypes for individual subjects according to various risk factor levels at baseline. To assess discrimination, the AUC (area under the ROC, or receiver operating curve) was obtained for progression within 5 years and progression within 10 years. In addition, an age-adjusted concordance or “C” statistic based on the curve was calculated to assess the probability that the risk score based on the group of risk factors in that model from a random progressor was higher than the corresponding risk score from a random non-progressor within the same 10-year age group [25]. Confidence limits were obtained and C statistics were compared between competing models [26].

To assess the added value of a model with genes vs. models with fewer or no genes, we calculated quintiles of risk score according to each model and cross-classified the quintile of risk score derived from one model by quintile of risk score derived from the other. We then ran a logistic regression of progression within 5 years (yes/no) on risk score quintile of the zero gene model (treated as a categorical variable with regression coefficients β_1_, β_2_, β_3,_ β_4_) and the 9 gene model risk score quintile, expressed as an ordinal variable with values 1 to 5 with regression coefficient γ. The odds ratio (OR) of progression to advanced AMD per one unit increase in quintile of the 9 gene model risk score, holding the risk score quintile of the zero gene model constant, was measured by exp (γ). A similar approach was used to assess the added value of the 9 gene model vs the 6 gene model.

## Results

Among 2765 individuals, there were 777 progressors to advanced stages of AMD in either eye, and 1988 non-progressors. Among the progressors, 416 progressed to GA in at least one eye and 527 progressed to NV in at least one eye. The mean ages (± SD) of progressors and non-progressors at baseline were 70.2 (±5.2) and 68.2 (±4.7).

**Table 1** displays the multivariate associations between baseline demographic, environmental and macular variables and incident advanced AMD. Increasing age, current smoking and higher BMI were related to progression. Advanced AMD in one eye and larger drusen size in the fellow eye, as well as larger drusen size in both eyes among individuals without advanced AMD at baseline were strongly related to higher rates of conversion from early and intermediate to advanced stages of AMD.

**Table 1:** Multivariate Associations between Baseline Demographic, Environmental, Macular Variables and Incidence of Advanced AMD.

		Progressors	Non-progressors		HR 95% CI*	p - value
Total patients:		(N = 777)	(N = 1988)			
Age (years)		N (%)	N (%)			
	<65	197 (25)	242 (12)		1.0 ref	
	65–74	476 (61)	1322 (66)		0.7 (0.6–0.9)	0.0007
	55–64	104 (13)	424 (21)		0.6 (0.5–0.7)	<0.0001
Sex						
	Female	425 (55)	1135 (57)		1.0 ref	
	Male	352 (45)	853 (43)		1.1 (0.9–1.2)	0.42
Education						
	≤ High School	309 (40)	624 (31)		1.0 ref	
	> High School	468 (60)	1364 (69)		0.9 (0.8–1.0)	0.07
Smoking						
	Never	300 (39)	1008 (51)		1.0 ref	
	Past	410 (53)	880 (44)		1.1 (1.0–1.3)	0.09
	Current	67 (9)	100 (5)		1.9 (1.4–2.4)	<0.0001
BMI						
	<25	227 (29)	672 (34)		1.0 ref	
	25–29	328 (42)	848 (43)		1.1 (0.9–1.3)	0.52
	30+	222 (29)	468 (24)		1.2 (1.0–1.5)	0.05
Advanced AMD in One Eye at Baseline						
	Neither Eye	522 (67)	1833 (92)		1.0 ref	
	Grade 4	49 (6)	6 (0.3)		7.1 (2.6–18.9)	<0.0001
	Grade 5	206 (27)	149 (7)		5.0 (1.9–12.6)	0.0008
Individuals with Advanced AMD in One Eye at Baseline: Largest Drusen Size in Non-advanced Eye (microns)						
	<63	6 (2)	46 (30)		1.0 ref	
	63–124	41 (16)	57 (37)		4.4 (1.9–10.4)	0.0007
	125–249	86 (34)	37 (24)		9.6 (4.2–22.0)	<0.0001
	≥250	122 (48)	15 (10)		16.6 (7.3–37.8)	<0.0001
Individuals Without Advanced AMD at Baseline: Size of Drusen (microns) in Each Eye						
	<63, <63	17 (3)	807 (44)	1.0 ref		
	63–124, <63	27 (5)	388 (21)	3.2 (1.8–6.0)		0.0002
	63–124, 63–124	35 (7)	171 (9)	8.4 (4.7–15.1)		<0.0001
	125–249, <63	21 (4)	127 (7)	7.3 (3.8–13.9)		<0.0001
	125–249, 63–124	64 (12)	155 (9)	15.3 (8.9–26.3)		<0.0001
	125–249, 125–249	88 (17)	93 (5)	30.1 (18.2–52.0)		<0.0001
	≥250, ≤124	24 (5)	25 (1)	29.6 (15.8–55.4)		<0.0001
	≥250, 125–249	92 (18)	41 (2)	49.7 (29.4–84.1)		<0.0001
	≥250, ≥250	154 (30)	26 (1)	75.0 (45.2–124.5)		<0.0001

HR = hazard ratio; CI = confidence interval.

* Controlling for all variables in the table.

The distributions of genetic variants among progressors and non-progressors are shown in **Table 2**. There were significant and positive associations between progression and the number of risk alleles for *CFH Y402H*, *CFH* rs1410996, *ARMS2/HTRA1* and *C3.* In addition there were protective effects for the minor alleles of *C2* and *CFB*. We found significant positive associations with risk alleles for *CFI*, *CETP*, and *HSPH1/B3GALTL,* and a significant protective association for the minor allele of *RAD51B*. Furthermore, there were borderline positive associations with *VEGFA* (P = .06) and borderline protective associations for *COL10A1* and *TIMP3* (P = .06). No significant associations with progression to advanced AMD were found for *LIPC*, *ABCA1*, *TNFRSF10A*, *APOC1/APOE*, *DDR1*, *SLC16A8*, *TGFBR1*, and *ADAMTS9* in these analyses.

**Table 2:** Distribution of Age-Related Macular Degeneration Genetic Variants Among Progressors and Non-Progressors.

Gene/Genotype		Progression			Gene/Genotype		Progression		
		Yes	No	p-value*			Yes	No	p-value*
Total patients N (%)		777 (28.1)	1988 (71.9)				777 (28.1)	1988 (71.9)	
*CFH*(Y402H)	TT	123 (16)	723 (36)	<0.0001	*LIPC*	CC	420 (54)	1036 (52)	0.25
	CT	343 (44)	922 (46)			CT	308 (40)	805 (40)	
	CC	311 (40)	343 (17)			TT	49 (6)	147 (7)	
*CFH*	TT	29 (4)	329 (17)	<0.0001	*ABCA1*	CC	443 (57)	1094 (55)	0.28
	CT	231 (30)	903 (45)			CT	290 (37)	765 (38)	
	CC	517 (66)	756 (38)			TT	44 (6)	129 (6)	
*ARMS2/HTRA1*(A69S)	GG	244 (31)	1164 (59)	<0.0001	*FRK/COL10A1*	CC	401 (52)	1046 (53)	0.73
	GT	368 (47)	701 (35)			CT	315 (41)	784 (40)	
	TT	165 (21)	123 (6)			TT	60 (8)	155 (8)	
*C2* (E318D)	GG	748 (96)	1827 (92)	<0.0001	*APOC1/APOE*	AA	568 (73)	1422 (72)	0.41
	CG/CC	29 (4)	161 (8)			AG/GG	209 (27)	566 (28)	
*CFB* (R32Q)	CC	715 (93)	1615 (84)	<0.0001	*TIMP3*	AA	712 (92)	1774 (89)	0.06
	CT/TT	56 (7)	318 (16)			AC/CC	65 (8)	214 (11)	
*C3* (R102G)	CC	386 (50)	1234 (62)	<0.0001	*TNFRSF10A*	TT	222 (29)	540 (27)	0.87
	CG	318 (41)	669 (34)			GT	373 (48)	1020 (51)	
	GG	73 (9)	84 (4)			GG	182 (23)	428 (22)	
*CFI*	CC	181 (23)	526 (26)	0.01	*ADAMTS9/AS2* **	CC	241 (31)	581 (29)	0.27
	CT	373 (48)	983 (50)			CT	375 (48)	964 (48)	
	TT	223 (29)	479 (24)			TT	161 (21)	443 (22)	
*CETP*	CC	310 (40)	880 (44)	0.003	*SLC16A8*	CC	493 (64)	1267 (64)	0.46
	AC	356 (46)	902 (45)			CT	241 (31)	641 (32)	
	AA	111 (14)	205 (10)			TT	43 (6)	80 (4)	
*COL8A1*	TT	594 (76)	1636 (82)	0.0004	*DDR1*	CC	546 (70)	1394 (70)	0.59
	CT	170 (22)	332 (17)			CT	218 (28)	540 (27)	
	CC	13 (2)	20 (1)			TT	13 (2)	54 (3)	
*COL10A1*	AA	292 (38)	715 (36)	0.06	*TGFBR1*	TT	463 (60)	1137 (57)	0.20
	AG	379 (49)	921 (46)			GT	271 (35)	722 (36)	
	GG	106 (14)	351 (18)			GG	43 (6)	129 (6)	
*CFH R1210C*	CC	769 (99)	1982 (99.7)	0.02	*VEGFA*	CC	150 (19)	451 (23)	0.06
	CT	8 (1)	6 (0.3)			CT	399 (51)	996 (50)	
*RAD51B*	AA	331 (43)	791 (40)	0.02		TT	228 (29)	540 (27)	
	AG	367 (47)	914 (46)		*HSPH1/B3GALTL*	TT	232 (30)	666 (34)	0.006
	GG	79 (10)	283 (14)			CT	369 (47)	963 (48)	
*HSPH1/B3GALTL*	TT	232 (30)	666 (34)	0.006		CC	176 (23)	359 (18)	
	CT	369 (47)	963 (48)						
	CC	176 (23)	359 (18)						

* Mantel-Haenszel Chi-Square.

** *ADAMTS9/ADAMTS9-AS2.*

**Table 3** displays the multivariate associations between incident AMD and the novel gene variants in two models: A) adjusted for demographic, environmental and macular variables, and B) controlling for the 6 genetic variants in 5 genes (referred to herein as the “6 gene model”), in addition to the non-genetic variables in Model A. In Model B, *COL8A1* (CC vs TT, HR = 1.9, P = .02, P trend = 0.04), *CFH R1210C* (HR 2.4, P = .02) and *RAD51B* (GG vs AA, HR 0.80, P = .04, P trend = 0.01), were significantly related to AMD progression to advanced stages independent of the other variables.

**Table 3:** Associations between New Age-Related Macular Degeneration Genetic Loci and Incidence of Advanced Age-Related Macular Degeneration, Controlling for Demographic, Environmental, Ocular and Genetic Factors.

Gene: SNP (Reference Genotype)/Genotype		Model A*			Model B‡		
		HR 95% CI	p-value	p-trend	HR 95% CI	p-value	p-trend
*CFI*:rs10033900 (CC)	CT	1.0 (0.9–1.2)	0.78		1.1 (0.9–1.3)	0.45	
	TT	1.1 (0.9–1.3)	0.48	0.47	1.1 (0.9–1.3)	0.38	0.39
*LIPC*:rs10468017 (CC)	CT	1.1(0.9–1.2)	0.51		1.0 (0.9–1.2)	0.65	
	TT	1.1(0.8–1.3)	0.42	0.35	1.1 (0.8–1.5)	0.40	0.41
*CETP:* rs3764261 (CC)	AC	1.1 (0.9–1.2)	0.48		1.0 (0.9–1.2)	0.56	
	AA	1.1 (0.9–1.4)	0.29	0.27	1.2 (0.9–1.4)	0.19	0.21
*ABCA1:* rs1883025 (CC)	CT	1.1 (0.9–1.2)	0.46		1.1 (0.9–1.2)	0.35	
	TT	1.0 (0.7–1.3)	0.75	0.78	0.9 (0.6–1.2)	0.32	0.95
*TIMP3:* rs9621532 (AA)	AC/CC	0.8 (0.6–1.0)	0.07	–	0.8 (0.6–1.0)	0.11	-
*COL8A1*:rs13095226 (TT)	CT	1.2 (1.0–1.4)	0.09		1.1 (0.9–1.3)	0.21	
	CC	1.9 (1.1–3.3)	0.02	0.02	1.9 (1.1–3.3)	0.02	0.04
*FRK/COL10A1:* rs1999930 (CC)	CT	1.1 (0.9–1.2)	0.45		1.0 (0.9–1.2)	0.93	
	TT	1.0 (0.7–1.3)	0.83	0.78	0.9 (0.7–1.2)	0.71	0.83
*COL10A1:* rs1064583 (AA)	AG	1.1 (0.9–1.3)	0.29		1.1 (0.9–1.3)	0.35	
	GG	0.8 (0.7–1.0)	0.13	0.36	0.8 (0.7–1.0)	0.10	0.28
*VEGFA:* rs943080 (CC)	CT	1.1 (0.9–1.3)	0.30		1.1 (0.9–1.3)	0.38	
	TT	1.0 (0.8–1.2)	0.72	0.54	1.0 (0.8–1.2)	0.96	0.82
*TNFRSF10A:* rs13278062 (TT)	GT	1.0 (0.8–1.2)	0.91		1.0 (0.8–1.2)	0.74	
	GG	1.2 (1.0–1.4)	0.11	0.14	1.1 (0.9–1.4)	0.22	0.27
*CFH R1210C*:rs121913059 (CC)	CT	1.4 (0.7–2.9)	0.31	–	2.4 (1.2–4.9)	0.02	–
*APOC1/APOE:* rs4420638 (AA)	AG/GG	1.0 (0.9–1.2)	0.80	–	1.0 (0.8–1.2)	0.94	–
*DDR1*:rs3094111 (CC)	CT	1.1 (0.9–1.3)	0.40		1.1 (0.9–1.3)	0.31	
	TT	0.9 (0.5–1.6)	0.82	0.54	1.1 (0.6–1.9)	0.77	0.32
*SLC16A8:* rs8135665 (CC)	CT	1.0 (0.9–1.2)	0.58		1.1 (0.9–1.2)	0.51	
	TT	1.2 (0.9–1.7)	0.23	0.25	1.2 (0.9–1.6)	0.29	0.27
*TGFBR1:* rs334353 (TT)	GT	1.0 (0.9–1.2)	0.85		1.0 (0.9–1.2)	0.98	
	GG	1.0 (0.8–1.4)	0.86	0.81	1.0 (0.7–1.4)	0.93	0.94
*RAD51B:* rs8017304 (AA)	AG	0.9 (0.7–0.99)	0.04		0.9 (0.7–0.99)	0.04	
	GG	0.7 (0.6–0.96)	0.02	0.007	0.8 (0.6–0.98)	0.04	0.01
*ADAMTS9/AS2* ** *:* rs6795735 (CC)	CT	1.0 (0.8–1.1)	0.89		1.0 (0.8–1.1)	0.73	
	TT	0.9 (0.8–1.3)	0.51	0.70	0.9 (0.8–1.1)	0.49	0.83
*HSPH1/B3GALTL:* rs9542236 (TT)	CT	1.2 (1.0–1.4)	0.03		1.1 (1.0–1.3)	0.17	
	CC	1.2 (1.0–1.5)	0.06	0.04	1.1 (0.9–1.3)	0.40	0.33

* Model A = Controlling for: age, gender, education, body mass index, smoking, 4 treatment groups,

baseline macular grade and drusen status.

‡ Model B = Controlling for: age, gender, education, body mass index, smoking, 4 treatment groups,

baseline macular grade, drusen status, *CFH*rs1410996, *CFH* Y402H, *ARMS2/HTRA1, C3, C2 and CFB.*

** *ADAMTS9/ADAMTS9-AS2.*

**Table 4** shows two models: 1) the 6 gene model in our previous prediction paper [22]–[24], with multivariate associations between incident advanced AMD and the genetic variants adjusted for demographic, environmental, and macular phenotypes, and 2) the 9 gene model (9 genetic loci in 7 genes, herein referred to as the “9 gene model”) with the addition of the 3 significant genetic loci identified in Table 3, mutually adjusted for each other as well as the other 6 genetic loci. We found independent effects of the variants in *COL8A1* (CC vs TT, HR = 2.0, P = .02, P trend = 0.04), *CFH R1210C* (CT vs CC, HR = 2.5, P = .01), and *RAD51B* (GG vs AA, HR = 0.80, P = .03, P trend = 0.01).

**Table 4:** Multivariate Associations Between Genes and Progression to Advanced Age-Related Macular Degeneration.

Gene: SNP/Genotype		6 Gene Model*			9 Gene Model‡		
		HR 95% CI	p-value	p-trend	HR 95% CI	p-value	p-trend
*CFH*:rs1061170 (Y402H)	TT	1.0 (ref.)			1.0 (ref.)		
	CT	1.1 (0.9–1.3)	0.63		1.1 (0.9–1.4)	0.40	
	CC	1.2 (0.9–1.5)	0.31	0.29	1.2 (0.9–1.5)	0.25	0.26
*CFH*:rs1410996	TT	1.0 (ref.)			1.0 (ref.)		
	CT	2.0 (1.3–3.0)	0.002		1.9 (1.2–2.9)	0.004	
	CC	2.4 (1.6–3.8)	<0.0001	0.0002	2.4 (1.5–3.7)	0.0001	0.0001
*ARMS2/HTRA1*:rs10490924(A69S)	GG	1.0 (ref.)			1.0 (ref.)		
	GT	1.3 (1.1–1.6)	0.001		1.3 (1.1–1.6)	0.0008	
	TT	1.8 (1.5–2.3)	<0.0001	<0.0001	1.9 (1.5–2.3)	<0.0001	<0.0001
*C2*:rs9332739(E318D)	GG	1.0 (ref.)			1.0 (ref.)		
	CG/CC	0.7 (0.5–1.0)	0.05	–	0.7 (0.5–1.0)	0.06	–
*CFB*:rs641153(R32Q)	CC	1.0 (ref.)			1.0 (ref.)		
	CT/TT	0.7 (0.5–0.9)	0.004	–	0.7 (0.5–0.9)	0.006	–
*C3*:rs2230199(R102G)	CC	1.0 (ref.)			1.0 (ref.)		
	CG	1.1 (1.0–1.3)	0.19		1.1 (1.0–1.3)	0.13	
	GG	1.4 (1.1–1.8)	0.01	0.01	1.4 (1.1–1.8)	0.009	0.006
*COL8A1*:rs13095226	TT	–	–		1.0 (ref.)		
	CT	–	–		1.1 (0.9–1.3)	0.21	
	CC	–	–		2.0 (1.1–3.5)	0.02	0.04
*CFH R1210C*:rs121913059	CC	–	–		1.0 (ref.)		
	CT	–	–		2.5 (1.2–5.3)	0.01	–
*RAD51B:* rs8017304	AA	–	–		1.0 (ref.)		
	AG	–	–		0.9 (0.7–1.0)	0.05	
	GG	–	–		0.8(0.6–0.97)	0.03	0.01

* 6 Gene Model = Controlling for: age, gender, education, body mass index, smoking, baseline macular grade, drusen status, 4 treatment groups, CFH rs1410996, CFHY402H, ARMS2/HTRA1, C2,C3 and CFB genes.

‡ 9 Gene Model = Controlling for: age, gender, education, body mass index, smoking, baseline macular grade, drusen status, 4 treatment groups and all genes in the table.

**Table 5** depicts similar analyses for progression to GA and NV separately. Associations with some genetic variants were somewhat stronger for NV than GA, although HR’s were in the same direction generally for both phenotypes, except for *RAD51B* which was not related to GA.

**Table 5:** 9 Gene Model for Geographic Atrophy and Neovascular Disease.

Gene: SNP/Genotype		Geographic Atrophy (N = 416)**			Neovascular Disease (N = 527)**		
		HR 95% CI*	p-value	p-trend	HR 95% CI*	p-value	p-trend
*CFH*:rs1061170 (Y402H)	TT	1.0 (ref)			1.0 (ref)		
	CT	1.08(0.78–1.5)	0.63		1.31(0.97–1.76)	0.08	
	CC	1.27(0.87–1.84)	0.22	0.19	1.26(0.9–1.76)	0.17	0.33
*CFH*:rs1410996	TT	1.0 (ref)			1.0 (ref)		
	CT	1.52(0.89–2.6)	0.13		1.77(1.01–3.1)	0.047	
	CC	1.69(0.96–2.99)	0.07	0.11	2.45(1.37–4.38)	0.002	<0.001
*ARMS2/HTRA1*:rs10490924(A69S)	GG	1.0 (ref)			1.0 (ref)		
	GT	1.39(1.1–1.75)	0.005		1.43(1.16–1.76)	<0.001	
	TT	1.74(1.3–2.32)	<0.001	<0.001	1.94(1.5–2.49)	<0.0001	<0.0001
*C2*:rs9332739(E318D)	GG	1.0 (ref)			1.0 (ref)		
	CG/CC	0.61(0.33–1.11)	0.11	0.10	0.78(0.5–1.21)	0.26	0.21
*CFB*:rs641153(R32Q)	CC	1.0 (ref)			1.0 (ref)		
	CT/TT	0.68(0.46–1.0)	0.050	0.06	0.62(0.44–0.88)	0.008	0.009
*C3*:rs2230199(R102G)	CC	1.0 (ref)			1.0 (ref)		
	CG	1.1(0.89–1.35)	0.39		1.13(0.94–1.36)	0.20	
	GG	1.26(0.87–1.83)	0.23	0.16	1.42(1.05–1.93)	0.026	0.021
*COL8A1*:rs13095226	TT	1.0 (ref)			1.0 (ref)		
	CT	1.05(0.83–1.34)	0.67		1.27(1.03–1.56)	0.025	
	CC	2.02(0.99–4.15)	0.055	0.24	1.61(0.79–3.24)	0.19	0.016
*CFH R1210C*:rs121913059	CC	1.0 (ref)			1.0 (ref)		
	CT	3.12(1.33–7.29)	0.009	0.006	3.09(1.33–7.19)	0.008	0.001
*RAD51B: rs8017304*	AA	1.0 (ref)			1.0 (ref)		
	AG	0.91(0.73–1.12)	0.36		0.90(0.76–1.08)	0.28	
	GG	0.96(0.68–1.34)	0.79	0.49	0.67(0.49–0.92)	0.013	0.016

* Controlling for: Age, gender, education, body mass index, smoking, baseline macular grade, drusen status, 4 treatment groups and all genes in the table.

** Some individuals progressed to Geographic Atrophy in one eye and Neovascular Disease in the fellow eye.

In **Table 6**, we estimated the AUC’s corrected for age for gene models predicting progression at 5 years and 10 years. For 5 year progression, the AUC’s were as follows: 0 gene model 0.873, 6 gene model 0.883, 9 gene model 0.884; P = 0.01 for 0 gene vs 9 gene model and P = 0.24 for 9 gene vs 6 gene model. Similarly, for 10 year progression, AUC’s were 0.898, 0.910, and 0.911 for the 0, 6, and 9 gene models, respectively, with P = 0.001 for the 9 vs 0 gene model comparison of the AUC’s but no significant difference in AUC’s for the 9 vs 6 gene model. Changes in AUC’s for the 9 gene vs 0 gene models were somewhat larger for NV than GA for both 5 and 10 year progression.

**Table 6:** Area Under the Curve Statistics for Progression to Advanced Age-Related Macular Degeneration, Geographic Atrophy and Neovascular Disease at 5 and 10 Years After Baseline.

	AUC (SE)	AUC (SE)	AUC (SE)	p-value	p-value
	0 Gene Model	6 Gene Model	9 Gene Model	6 vs 9 Gene Model	0 vs 9 Gene Model
5 Year					
All advanced AMD	0.873 (0.009)	0.883 (0.008)	0.884 (0.008)	0.24	0.01
Geographic Atrophy	0.886 (0.012)	0.892 (0.012)	0.893 (0.012)	0.64	0.12
Neovascular Disease	0.860 (0.012)	0.875 (0.011)	0.876 (0.011)	0.30	0.02
10 Year					
All advanced AMD	0.898 (0.007)	0.910 (0.006)	0.911 (0.006)	0.56	0.001
Geographic Atrophy	0.914 (0.009)	0.921 (0.008)	0.920 (0.008)	0.90	0.02
Neovascular Disease	0.879 (0.009)	0.896 (0.009)	0.897(0.009)	0.43	0.001

SE = Standard Error.

**Figure 1:**
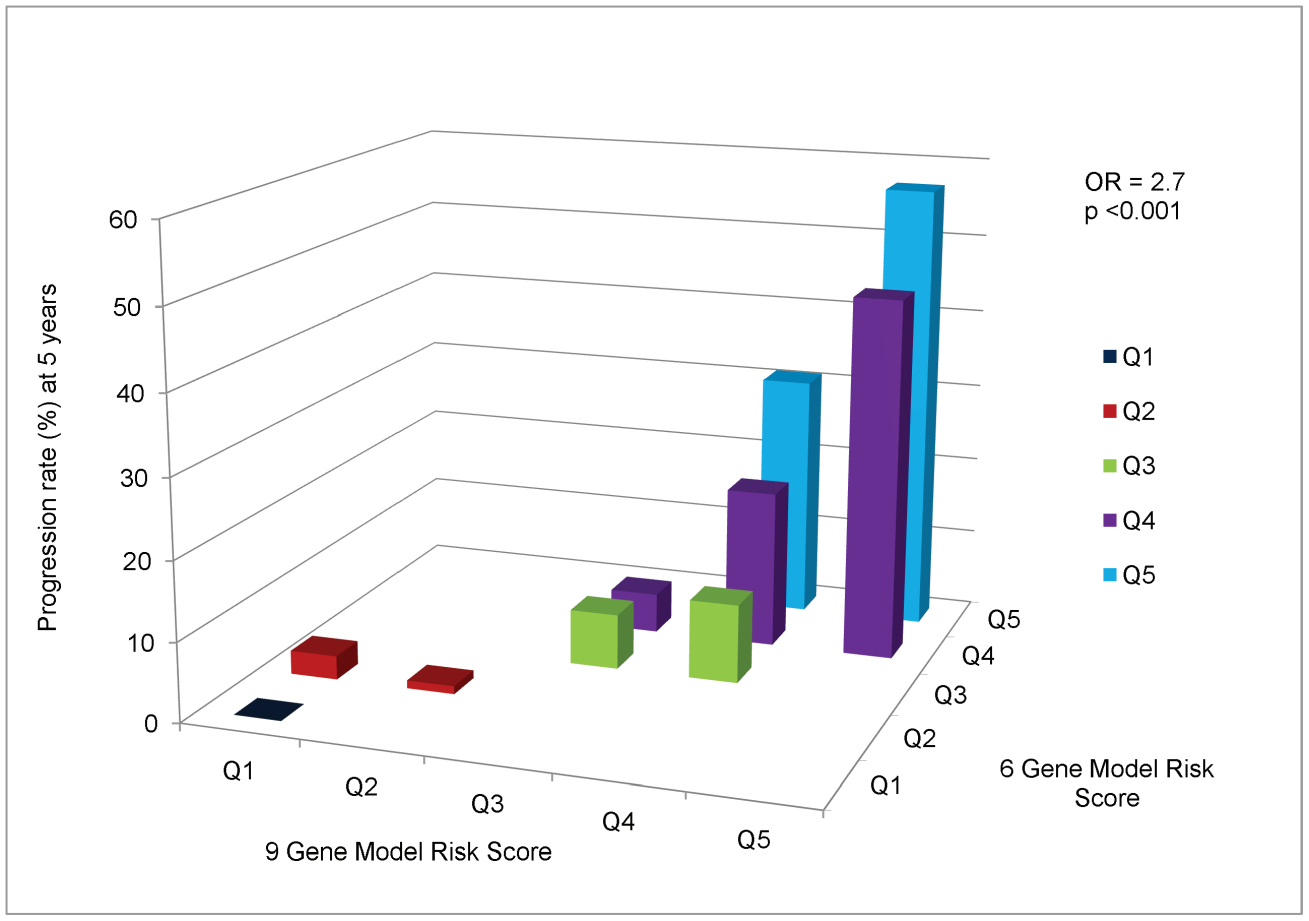
Cross-Classification of Progression Rates to Advanced AMD: 9 Gene Model vs 6 Gene Model. Cross-classification of subjects by risk score quintile for 9 and 6 gene models, with estimated progression rates for each combination of a 9 gene quintile by 6 gene quintile. OR = odds ratio of progression per one quintile increase in 9 gene model, holding 6 gene model quintile constant.

Previous studies in a variety of settings have revealed that the AUC is a relatively insensitive statistic for identifying improvement in model fit [27]. An alternative approach is provided by reclassification models where risk is cross-classified simultaneously for level of risk predicted by 2 different competing models. For this purpose we cross-classified subjects by risk score derived from the models and divided into quintiles (Q) and estimated rates of progression for each combination of a 6 gene risk score quintile by a 9 gene risk score quintile. The results are displayed in **Figure 1**. Qualitatively it appears, especially for high risk individuals (risk score Q4 and Q5), while holding the 6 gene model risk score quintile constant, there is an increasing progression rate as the 9 gene risk score quintile increases. Little additional information is provided for low risk individuals (Q1 and Q2). Overall, the OR per risk score quintile increase in the 9 gene model controlling for the 6 gene model risk score quintile was 2.7 (95% CI 1.7–4.4) P<0.001, indicating that significant additional information regarding progression is provided by the 9 gene model compared to the 6 gene model.

**Figure 2:**
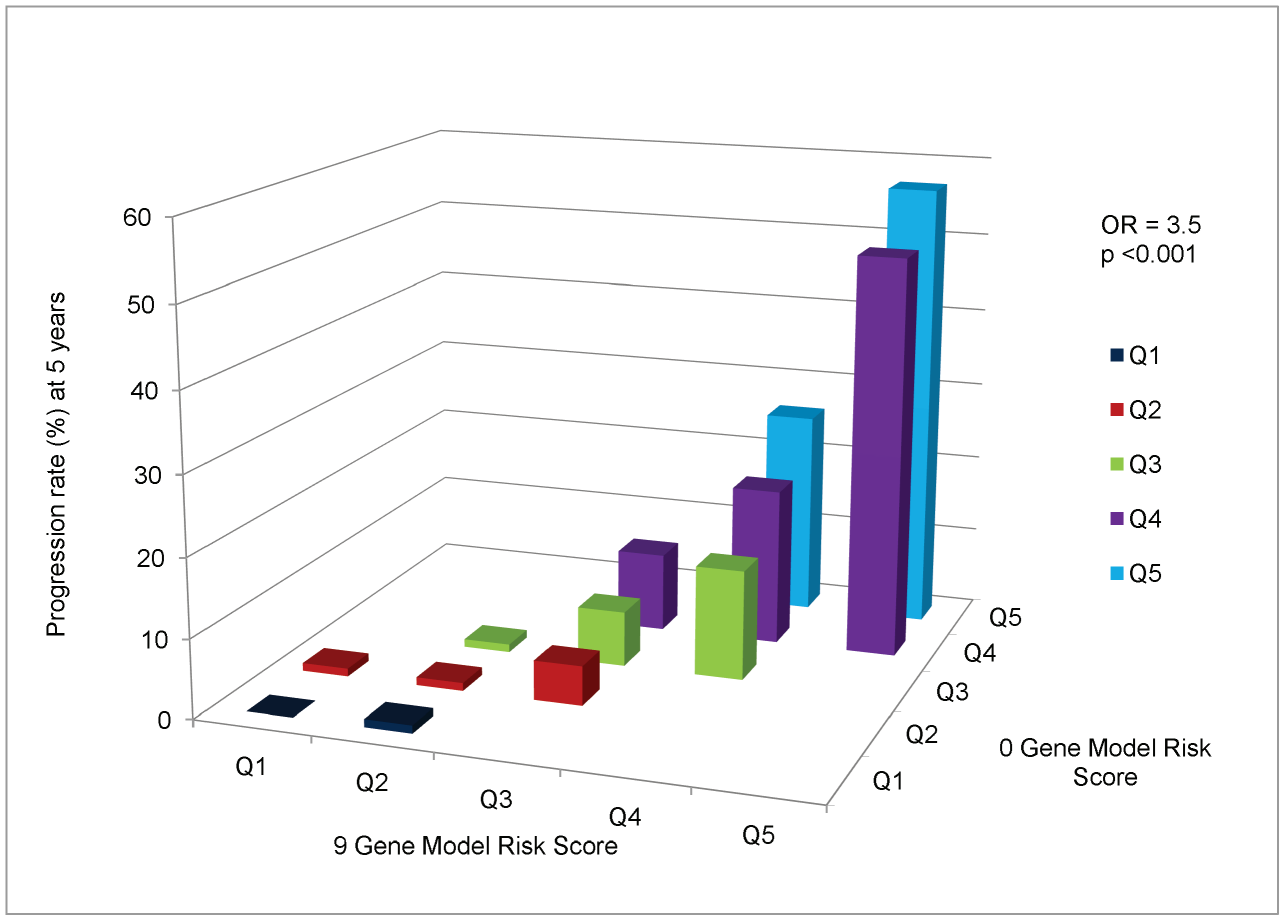
Cross-Classification of Progression Rates to Advanced AMD: 9 Gene Model vs 0 Gene Model. Cross-classification of subjects by risk score quintile for 9 and 0 gene models, with estimated progression rates for each combination of a 9 gene quintile by 0 gene quintile. OR = odds ratio of progression per one quintile increase in 9 gene model, holding 0 gene model quintile constant.

Given the ongoing discussion of the value in adding genetic information to predictive models, we also compared the model with 9 genetic loci to the model without genes (0 gene model) as shown in **Figure 2**. There are larger differences between the 9 gene vs the 0 gene model than between the 9 gene vs 6 gene models. There are large differences in progression rate as the 9 gene model risk score quintile increases, when holding the 0 gene model risk score quintile constant, for the highest risk individuals (i.e. Q4 and Q5 in the 0 gene model). For intermediate risk individuals (Q2 and Q3) for the 0 gene model, there are also discernible increases in progression rates as the 9 gene model risk score quintile increases. However, little added value is apparent for the lowest risk quintile (Q1) for the 9 gene loci vs 0 gene model. The OR per quintile increase in the 9 gene model controlling for the 0 gene model was 3.5 (95% CI 2.6–4.6), P<0.001, indicating that significant additional information is obtained by including genes in the predictive model.

**Figure 3:**
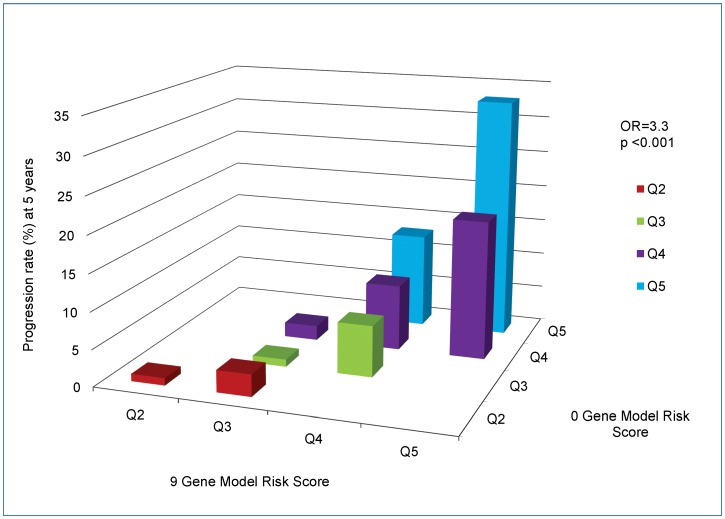
Cross-Classification of Progression Rates to Geographic Atrophy: 9 Gene Model vs 0 Gene Model. Cross-classification of subjects progressing to Geographic Atrophy by risk score quintile for 9 and 0 gene models, with estimated progression rates for each combination of a 9 gene quintile by 0 gene quintile. OR = odds ratio of progression per one quintile increase in 9 gene model, holding 0 gene model quintile constant.

**Figure 4:**
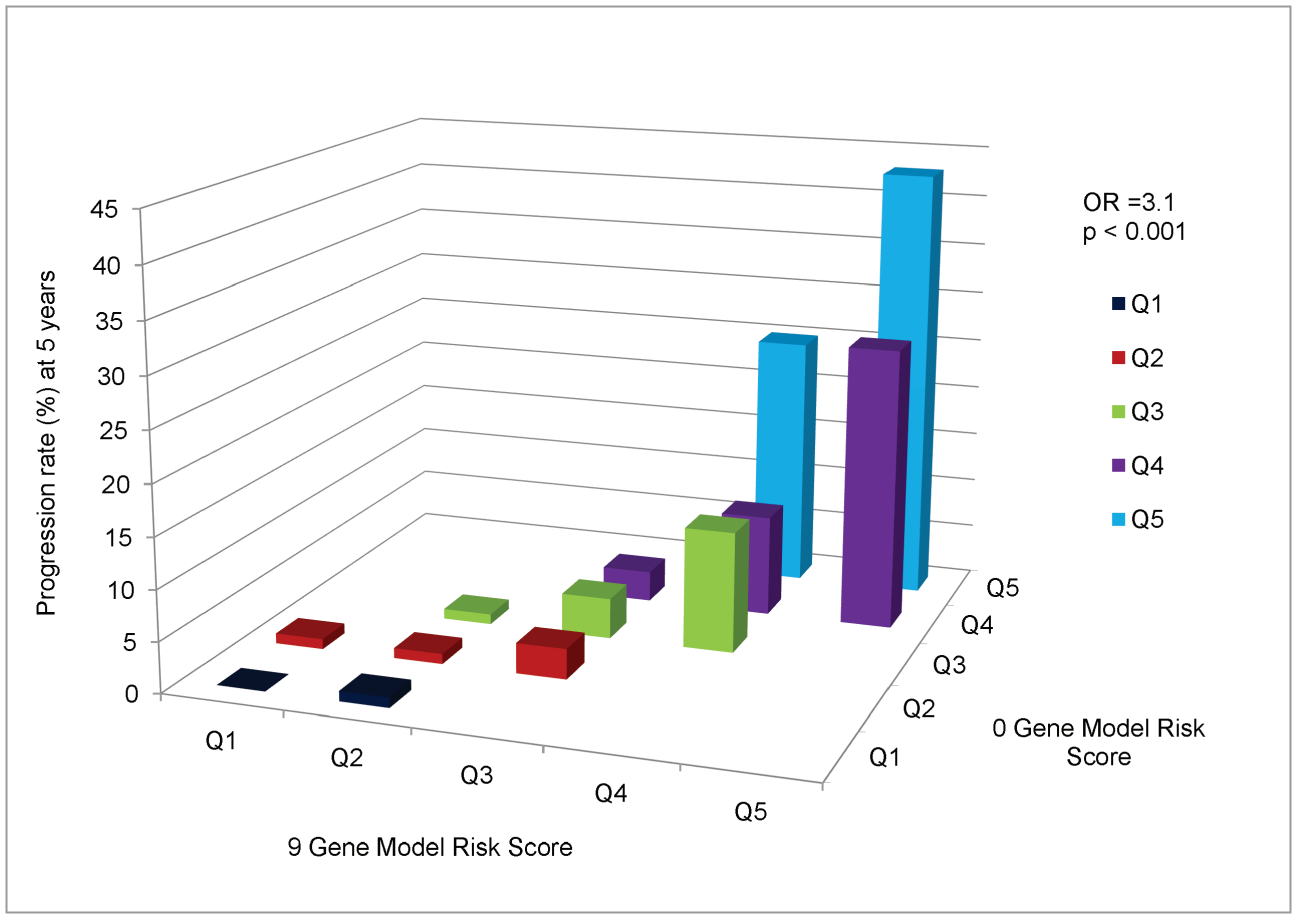
Cross-Classification of Progression Rates to Neovascular Disease: 9 Gene Model vs 0 Gene Model. Cross-classification of subjects progressing to Neovascular Disease by risk score quintile for 9 and 0 gene models, with estimated progression rates for each combination of a 9 gene quintile by 0 gene quintile. OR = odds ratio of progression per one quintile increase in 9 gene model, holding 0 gene model quintile constant.

In **Figures 3** **and** **4**, we depict the comparison of the 9 gene vs 0 gene models for progression at 5 years to GA and NV separately. The major incremental precision obtained from including 9 genes in the prediction model is obtained from subjects at high risk in the 0 gene model (i.e. Q4 and Q5), with smaller increments for intermediate risk subjects (Q3) and little benefit for low risk individuals (Q1 and Q2). The OR’s are 3.3, P<0.001 for GA (**Figure 3**), and 3.1, P<0.001 for NV (**Figure 4**).

## Discussion

To our knowledge this is the first report on the independent associations between R1210C in *CFH*, *COL8A1* and *RAD51B* and progression to advanced AMD, controlling for all known AMD genetic loci. The AUC’s for the 9 gene vs 0 gene models were significantly different but were similar for the 9 and 6 gene models. To further discriminate between the two models which included genes, we used a re-classification approach, which is novel for AMD risk models, but has been used in other settings [27]. Cross classifying quintile of risk in the 9 gene by 6 gene models, holding the 6 gene model constant, demonstrated an increased risk of progression in the 4^th^ and 5^th^ quintiles of the risk score, indicating that incorporating a larger number of independent AMD genetic loci enhanced predictive power. Using a similar approach, larger increases in predictive accuracy were apparent for the model with 9 genes compared to none.

Our first prediction models for advanced AMD beginning in 2006 included only genes [11], a model with environmental and demographic variables only (AUC 0.62), and a model that included *CFHY402H* genotype (AUC 0.74) [28]. These early models demonstrated the importance of genetic variants in predicting AMD risk. When *ARMS2/HTRA1* was added to the genetic model with *CFHY402H* along with demographic and environmental factors, the AUC increased moderately to 0.78 [29]. The first multi-gene prediction model with 6 loci in 5 genes in addition to demographic and environmental factors, increased the C statistic to 0.83, adding to the evidence that genetic susceptibility plays a large role in predicting AMD risk [22]. The addition of macular phenotype and baseline AMD grade to the genetic and environmental models increased the AUC to 0.89 [23]. Other prediction models include our Markov model of transitions within different stages of AMD and inclusion of plasma complement levels in the model [30], [31]. The Markov model included *CFH*, *ARMS2*, *C2*, *CFB*, *C3*, *CFI* and genes in the cholesterol and collagen pathways (*LIPC, CETP, COL8A1*) in addition to drusen phenotypes, demographic, and environmental characteristics. The 5 year AUC in that model was 0.88 [30]. The addition of complement plasma markers to a model in a case-control study, together with 6 complement pathway loci, *ARMS2/HTRA1*, demographic and environmental characteristics increased the C-statistic to 0.94 [31].

AUC is a reasonable measure of discrimination for a risk prediction rule and by definition is based on the relative order of risk between progressors and non-progressors. However, it does not take into account the magnitude of the differences in risk. Thus, it does not provide all the information in determination of risk. In populations dominated by low risk individuals, there are ways to re-classify higher risk subjects to obtain a more accurate risk profile. Cook et al. demonstrated this in the Women’s Health Study population assessing cardiovascular risk in models with and without hsCRP [27]. In that study, hsCRP had little effect on the C-statistic. However, if one cross-classifies subjects by risk level with and without hsCRP, individuals in the low and medium risk groups (5–10% and 10–20% 10 year Framingham risk) were re-classified (21% and 19% of the time, respectively) into different risk groups [27]. Rosner et al. used a similar approach for cross-classifying breast cancer risk scores with and without estradiol [32]. Similar to the hsCRP study, the AUC did not increase substantially with the addition of estradiol. However, when risk deciles in the estradiol and non-estradiol models were cross-classified, there was an estimated 67% increase in breast cancer incidence for an increase of one decile of risk for the estradiol model holding the non-estradiol model decile constant [32]. In our study of macular degeneration, 5–10% of subjects were reclassified into a different quintile for the 9 gene vs 6 gene models, and 15–30% of subjects were reclassified for the 9 vs 0 gene models, indicating an improvement in model accuracy with the addition of genetic variants.

The mechanisms by which *CFH* R1210C, *COL8A1*, and *RAD51B* genes are related to the development and progression of advanced AMD are being explored. The R1210C mutation has been shown to compromise portions of the complement cascade resulting in defective binding to C3d, C3b, heparin and endothelial cells [3]. The *COL8A1* gene, encodes one of the two alpha chains of type VIII collagen, a major component of the multiple basement membranes in the eye, including Bruch’s membrane and the choroidal stroma [4], [33]. The protein encoded by *RAD51B* is a member of the RAD51 protein family, and is essential for DNA repair mechanisms. This gene is also involved in cell cycle delay and apoptosis [34].

## Conclusions

We have presented a model with 9 common and rare predictive genetic loci for progression to advanced stages of AMD that adds more predictive power than either a model with 6 common genetic loci or a model without any genetic information. New rare and highly penetrant loci in addition to the rare variant *CFH* R1210C [3], [35] and several common loci included here, may further improve the accuracy of AMD risk models. Our models can be used for clinical research such as selecting individuals at high risk for increased surveillance and for inclusion in clinical trials of new therapies [23], and for assessing different responses to AMD treatments based on the risk score.
